# Research progress on ATP-citrate lyase and its inhibitors: a metabolic-epigenetic perspective

**DOI:** 10.1080/14756366.2026.2744392

**Published:** 2026-10-14

**Authors:** Qinglin Liu, Hongbin Sun, Liang Dai

**Affiliations:** ^a^ Jiangsu Key Laboratory of Drug Discovery for Metabolic Disease, State Key Laboratory of Natural Medicines, China Pharmaceutical University, Nanjing, China; ^b^ Chongqing Innovation Institute of China Pharmaceutical University, Chongqing, China

**Keywords:** ATP-citrate lyase, ACLY inhibitors, structural biology, structure-activity relationships, drug discovery

## Abstract

ATP-citrate lyase (ACLY) is a key enzyme in cellular metabolism that catalyses the cleavage of citrate to produce acetyl coenzyme A (acetyl-CoA), thereby bridging energy metabolism and epigenetic regulation. Acetyl-CoA serves as a common substrate for de novo lipogenesis (DNL) and histone acetylation. Aberrant ACLY activity is closely associated with various pathological conditions, including metabolic diseases, Immune-mediated inflammatory diseases (IMIDs), cancer, and neurodegenerative diseases, positioning ACLY as an important therapeutic target for these diseases. However, existing inhibitors face challenges such as insufficient selectivity and limited clinical efficacy and off-target effects. This review systematically summarises the molecular mechanisms and pathological significance of ACLY within the overarching framework of its dual metabolic-epigenetic functions, from three perspectives, namely its structure and function, disease relevance, and inhibitor development and optimisation strategies, aiming to provide a theoretical foundation for precision therapies targeting ACLY.

## Introduction

ATP-citrate lyase (ACLY) is an important rate-limiting enzyme connecting glucose metabolism and lipid synthesis, catalysing the cleavage of citrate in the cytoplasm to produce acetyl-CoA and oxaloacetate (OAA)^1^^,^^2^. As the primary carbon source for the synthesis of fatty acids, cholesterol, and isoprenoids, acetyl-CoA substantially influences the rate of DNL^3^^,^^4^. More importantly, histone acetylation as an important epigenetic regulatory mechanism, is highly dependent on the availability of acetyl-CoA in the nucleus^5^. Therefore, ACLY is regarded as a “metabolic-epigenetic crosstalk hub” linking metabolic status to gene expression programs^6^^,^^7^. Dysregulation of this hub can lead to various chronic diseases, including metabolic diseases^8^, IMIDs^9^, cancer^10^, and neurodegenerative diseases^11^. Under physiological conditions, normal ACLY activity contributes to metabolic homeostasis^12^; under pathological conditions, the enzyme is significantly upregulated in multiple metabolic diseases (e.g., hyperlipidaemia) and malignancies (e.g., liver, lung, and breast cancer), driving lipid metabolic reprogramming and thereby supporting tumour cell membrane biogenesis, signal transduction, and energy storage^13–15^.

Given that ACLY possesses dual functions in metabolic substrate supply and epigenetic regulation, its inhibition exerts complex effects. On the one hand, inhibiting ACLY may activate acetate metabolism and compensatory pathways such as those mediated by acetyl-CoA synthetase 2 (ACSS2) in normal cells or under specific metabolic conditions to maintain baseline levels of acetyl-CoA and histone acetylation, thus partially diminishing the expected efficacy of the inhibitors^16^. On the other hand, this inhibition downregulates histone acetylation levels in tumour cells, thereby suppressing oncogene expression and tumour proliferation, demonstrating therapeutic potentia in specific tumour contexts^17^^,^^18^. In addition, acetyl-CoA derived from ACLY not only mediates the aforementioned transcriptional regulation but also drives the acetylation of downstream non-histone proteins (e.g., transcription factors), thereby extending epigenetic effects to the functional regulation of signalling proteins^19^^,^^20^. These findings indicate that the design of ACLY inhibitors should not only consider their metabolic regulatory effects but also systematically evaluate their impact on the epigenetic network to achieve more precise therapeutic targeting.

Previous reviews have summarised the roles of ACLY from different perspectives, covering its structure and enzymatic properties, inhibitor development in specific disease fields, and an overview of its biology and pharmacology^21–26^. However, from a metabolic-epigenetic perspective, few existing reviews have systematically addressed the research progress of ACLY inhibitors within a dual-function framework. Against this background, this review addresses three aspects: the structure and function of ACLY, its pathological relevance, and advances in inhibitor research, systematically reviewing the recent findings and core challenges under the metabolic-epigenetic dual framework, aiming to provide a reference for subsequent drug development and clinical translation.

## Structure and catalytic mechanism of ACLY

### Gene location and full-length structure

ACLY is primarily localised in the cytoplasm and enriched in the perinuclear endoplasmic reticulum, but it can also be detected in the nuclei of certain cell types^5^^,^^27^. Human ACLY (hACLY) is encoded by the ACLY gene on chromosome 17q21 and functions as a homotetramer^28^, sharing ∼98% sequence identity with its rat ortholog. Each subunit comprises 1,101 amino acid residues (∼120 kDa) and contains six domains. These domains are functionally ordered from the N- to the C-terminus as follows: domains 3, 4, 5, 1, 2, and the C-terminal citrate synthase (CS) domain. Domains 3 and 4 constitute the ATP-grasp fold (ATP-binding region), domain 5 forms the citrate-binding site, domain 1 mediates CoA binding, and domain 2 harbours the critical catalytic residue His760, whose phosphorylation is central to activity regulation. The C-terminal CS domains collectively form the central rigid core of the tetramer (Figure 1A)^29^.

**Figure 1.**
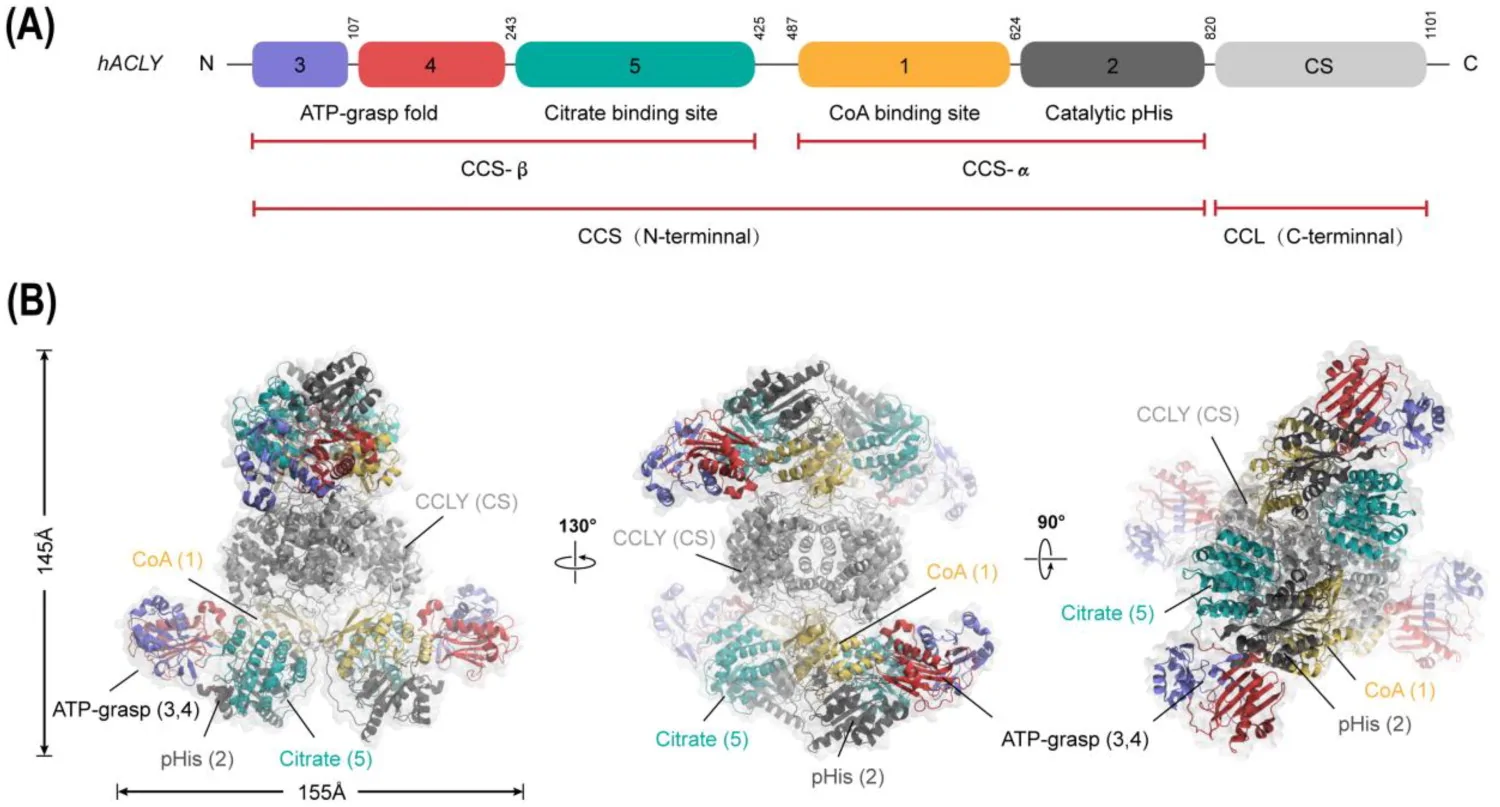
(A) Architecture of hACLY. (B) Overall and detailed views of the hACLY cryo-EM structure (PDB ID: 6O0H). The monomer is coloured by domain. Figure was created by the author using PyMOL and Figdraw software, based on references^29^^–^^31^.

The quaternary structure of ACLY adopts a “butterfly-shaped” conformation. The four subunits form a central core via their CS domains, with the N-terminal domains arranged on either side with D2 symmetry; the two monomers on one side are nearly perpendicular to those on the other (Figure 1B)^2^^,^^30^. From a modular perspective, the N-terminal domains 3, 4, and 5 constitute the citrate lyase citrate-CoA synthase (CCS) β-subunit, while domains 1 and 2 constitute the CCS α-subunit; these associate closely to form the CCS module. The C-terminal CS domain alone constitutes the citrate-CoA lyase (CCL) module (Figure 1A). The buried surface area for each protomer within the tetramer is approximately 7000 Å^2^, contributing to structural stability. The N-terminal segments are primarily distributed peripherally, especially around the ATP-grasp domains, with the overall maximum dimension of the complex approaching 150 Å (Figure 1B)^30^.

Notably, the CS domain of ACLY shares only ∼20% sequence identity with classical citrate synthase and lacks a complete set of catalytic residues. It therefore lacks citrate synthase activity^31^, but rather functions principally as a citryl-CoA lyase.

### Biological functions of ACLY

ACLY is highly expressed in tissues with active lipid metabolism, such as the liver, kidney, brain, pancreatic β-cells, and adipocytes. As a major source of cytoplasmic acetyl-CoA, it serves as a major hub connecting mitochondrial energy metabolism with cytoplasmic anabolism^32^. The catalytic reaction of ACLY follows an ordered mechanism. Mg^2+^ and ATP first bind to ACLY, and the γ-phosphate group of ATP is transiently transferred to the catalytic residue His760, forming a transient phosphohistidine intermediate; after ADP leaves, citrate and CoA bind successively, and the phosphate group subsequently transfers from phosphohistidine to the hydroxyl group of citrate, generating the high-energy intermediate citryl-phosphate^33^. coenzyme A then attacks citryl-phosphate form citryl-CoA, ACLY undergoes a ∼35 Å conformational change to cleave it into acetyl-CoA and OAA^31^, while ATP is ultimately hydrolysed to ADP and Pi (Figure 2)^34^^,^^35^. Acetyl-CoA as a primary substrate of acetyl-CoA carboxylase (ACC), drives fatty acid synthase (FASN)-mediated carbon chain elongation, thereby establishing a tightly coupled functional relationship among ACLY, ACC, and FASN^36^. Moreover, acetyl-CoA serves as the initiating unit of the mevalonate pathway in cholesterol synthesis, and its availability substantially influences the flux of 3-hydroxy-3-methylglutaryl-coenzyme A (HMG-CoA) reductase, ultimately leading to the production of cholesterol and steroid hormones via the mevalonate pathway^37^. The other product, oxaloacetate, can be converted to malate by malate dehydrogenase^38^, or further converted to pyruvate by malic enzyme with concomitant production of NADPH, thereby participating in gluconeogenesis, replenishing tricarboxylic acid (TCA) cycle intermediates, and maintaining cytoplasmic redox balance.

**Figure 2.**
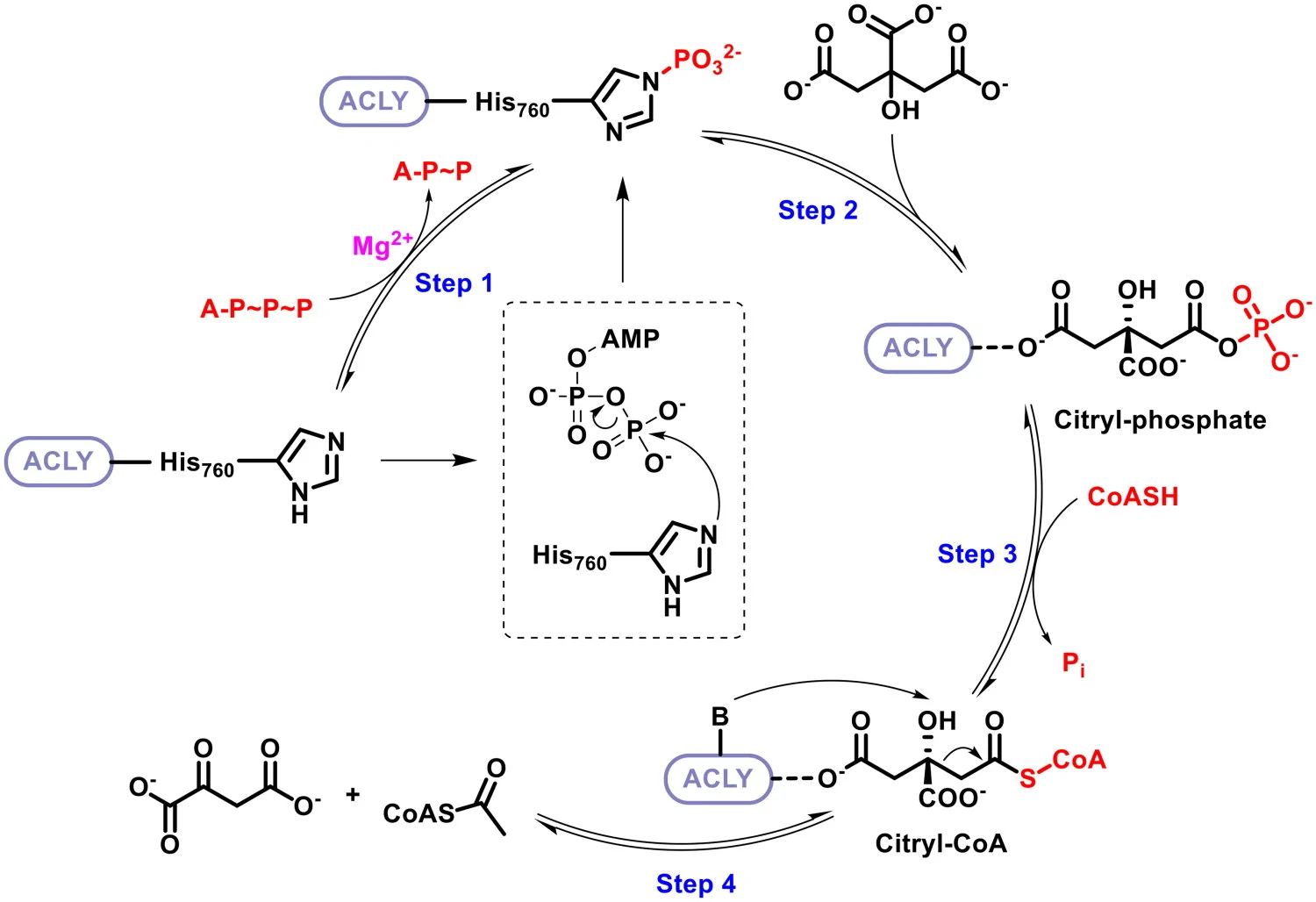
Mechanism of the ACLY-catalysed reaction. Figure was created by the author using ChemDraw software, redrawn based on reference^22^.

Beyond serving as a precursor for anabolism, acetyl-CoA is an important substrate for nuclear epigenetic modifications^39^. Under various stimuli (e.g., DNA damage, inflammatory signals, hypoxia, starvation, and chemotherapy), ACLY promotes its nuclear translocation through multi-site phosphorylation at Thr447/Ser451/Ser455 mediated by kinases such as protein kinase B (AKT) (this modification represents a signal-dependent regulatory phosphorylation, which is distinct from the transient phosphohistidine intermediate formed during the His760 catalytic cycle), thereby establishing a local “metabolic microenvironment” near chromatin^5^^,^^40^. ACLY interacts with histone acetyltransferases such as p300, histone acetyltransferase 1 (HAT1), or lysine acetyltransferase 2 A (KAT2A) to form functional complexes, catalysing the local generation of concentrated acetyl-CoA pools from citrate and coenzyme A in the vicinity of chromatin and thereby supplying substrates for both histone and non-histone protein acetylation^16^^,^^41^. Thus, ACLY not only bridges catabolism and biosynthesis but also closely links cellular metabolism with epigenetic regulation.

Notably, the regulation of ACLY is integrated into complex metabolic networks, exhibiting marked feedback and compensatory features^42^^,^^43^. For example, hepatocyte-specific knockdown of ACLY reduces acetyl-CoA levels but may stabilise the transcription factor sterol response element binding protein-1c (SREBP-1c), leading to transcriptional upregulation of compensatory molecules such as ACSS2, thereby partially sustaining lipid synthesis flux^44^. Furthermore, inhibition of ACLY activity decreases cholesterol synthesis, which can be compensated by upregulation of low-density lipoprotein (LDL) receptor expression through the sterol response element binding protein 2 (SREBP2) pathway, further reflecting the feedback regulatory nature of the metabolic network^45^. These findings reveal the considerable compensatory capacity of cells to maintain metabolic homeostasis and highlight the complexity of intervening in lipid synthesis by targeting a single metabolic node (Figure 3).

**Figure 3.**
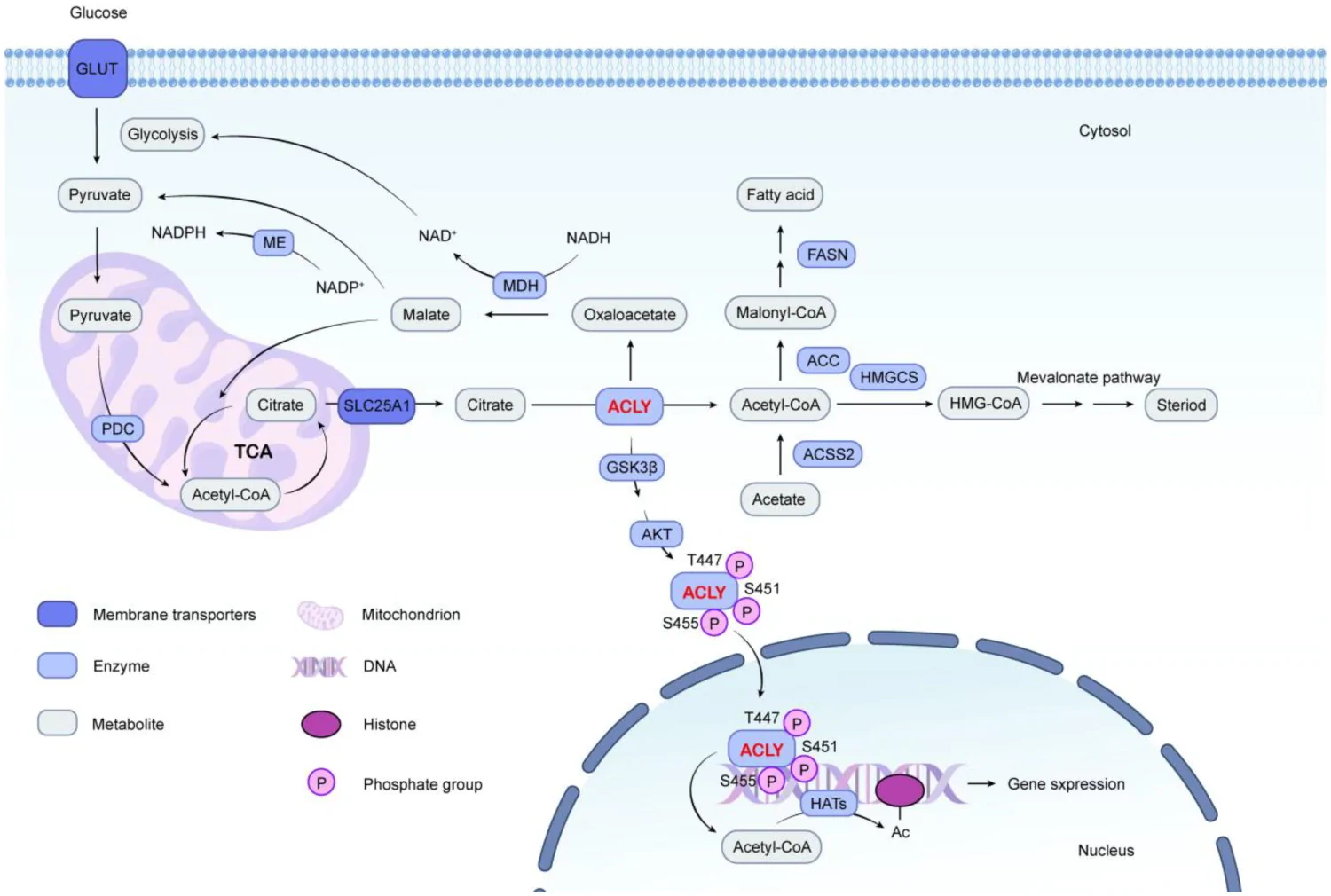
Schematic overview of mechanism of action of ACLY. Figure was created by the author using Figdraw software, based on references^36^^–^^45^.

## Role of ACLY in diseases

### Metabolic diseases

The pathological progression of obesity-related metabolic diseases involves the interaction between glucose-lipid metabolic disorders and chronic inflammation^46^^,^^47^. As an important rate-limiting enzyme linking glucose metabolism and lipid synthesis, ACLY plays an important role in various metabolic diseases through its catalytic product acetyl-CoA, which possesses dual properties of metabolic substrate supply and epigenetic regulation^43^. However, the predominant roles of ACLY’s metabolic and epigenetic functions may differ across different diseases and pathological stages^48^. The next section will focus on metabolic dysfunction-associated steatohepatitis (MASH)^3^^,^^49^, hyperlipidaemia, and atherosclerosis^48^, systematically elucidating how ACLY drives disease progression through the metabolic-epigenetic pathway and exploring its differential regulatory mechanisms under distinct pathological contexts. Elucidating these mechanisms will deepen our understanding of obesity-related metabolic dysfunction and provide new targets for the precise intervention of associated complications.

#### MAFLD/MASH

MASH is one of the most common chronic progressive liver diseases worldwide, with a global adult prevalence of approximately 5% and an upward trend, and has become a major cause of liver cirrhosis and hepatocellular carcinoma^50^^,^^51^. During its pathological progression, ACLY exhibits a dual role, with a context-dependent shift between “metabolic-driven” and “epigenetic-driven” functions^52^. In the early stage (simple steatosis), ACLY acts as an important rate-limiting enzyme catalysing citrate cleavage to produce acetyl-CoA, showing increased activity and predominantly functioning in lipid synthesis. Acetyl-CoA serves not only as a major carbon source for DNL but also as the precursor for palmitic acid and triglycerides, which directly drive hepatic lipid accumulation^13^^,^^53^. Meanwhile, cholesterol synthesis initiated by acetyl-CoA may activate hepatic stellate cells via the Hedgehog signalling pathway, potentially contributing to subsequent fibrosis progression^54^^,^^55^. Notably, acetyl-CoA generation is primarily derived from two sources: the citrate pathway catalysed by ACLY and a compensatory pathway catalysed by ACSS2 using acetate derived from the gut microbiota^42^. The intervention strategy targeting ACLY has been evaluated in preclinical and clinical settings at the metabolic regulation level. Preclinical studies have shown that single-target inhibition of ACLY can conditionally upregulate the ACSS2-mediated compensatory pathway^42^, potentially creating a feed-forward loop in lipid synthesis; in contrast, the small molecule EVT0185 (compound **19**, Figure 4A), which dually inhibits ACLY and ACSS2, effectively blocks this compensation^54^. In addition, preliminary progress has been made in the clinical translation of ACLY inhibitors. BGT-002 (compound **18**, 326E, Figure 4A), as the first ACLY/peroxisome proliferator-activated receptor α (PPARα) dual-target modulator to enter MASH clinical trials, has been reported to be well tolerated in MASH patients with once-daily oral administration (NCT06491576). The preliminary response rate of liver fat content reduction greater than 30% after 28 days of treatment was 50% (25% in the placebo group), accompanied by improvements in serum transaminase and γ-GGT levels^56^. The above clinical evidence mainly validates the intervention value of ACLY in the metabolic regulation of MASH, while as the disease progresses to fibrosis and inflammation stages, the epigenetic regulatory function of ACLY may become increasingly relevant. High ACLY expression in MASH liver tissue enhances the nuclear acetyl-CoA pool, promotes histone acetylation, and may consequently upregulates transcription of various rate-limiting lipogenic enzymes and pro-inflammatory cytokines (e.g., TNF-α, IL-6 and IL-1β) (Figure 5A)^13^^,^^57^. Chromatin immunoprecipitation and quantitative histone acetylation studies have demonstrated that ACLY inhibition attenuates lipid accumulation and oxidative damage in human hepatocytes triggered by TNF-α and reduces inflammatory cytokine secretion. Furthermore, *ACLY* mRNA levels in macrophages derived from MASH patient peripheral blood mononuclear cells were significantly higher than those in healthy controls; moreover, the natural ACLY inhibitor hydroxycitrate acid (compound 2, HCA, Figure 6) combined with red wine extract markedly downregulated ACLY expression and IL-6/IL-1β production^13^. These findings indicate that ACLY not only supplies lipid synthesis substrates via metabolic pathways but also regulates histone acetylation through nuclear acetyl-CoA, thereby shaping the transcriptional output of inflammation-related genes and potentially establishing a feed-forward amplification loop between metabolism and epigenetics.

**Figure 4.**
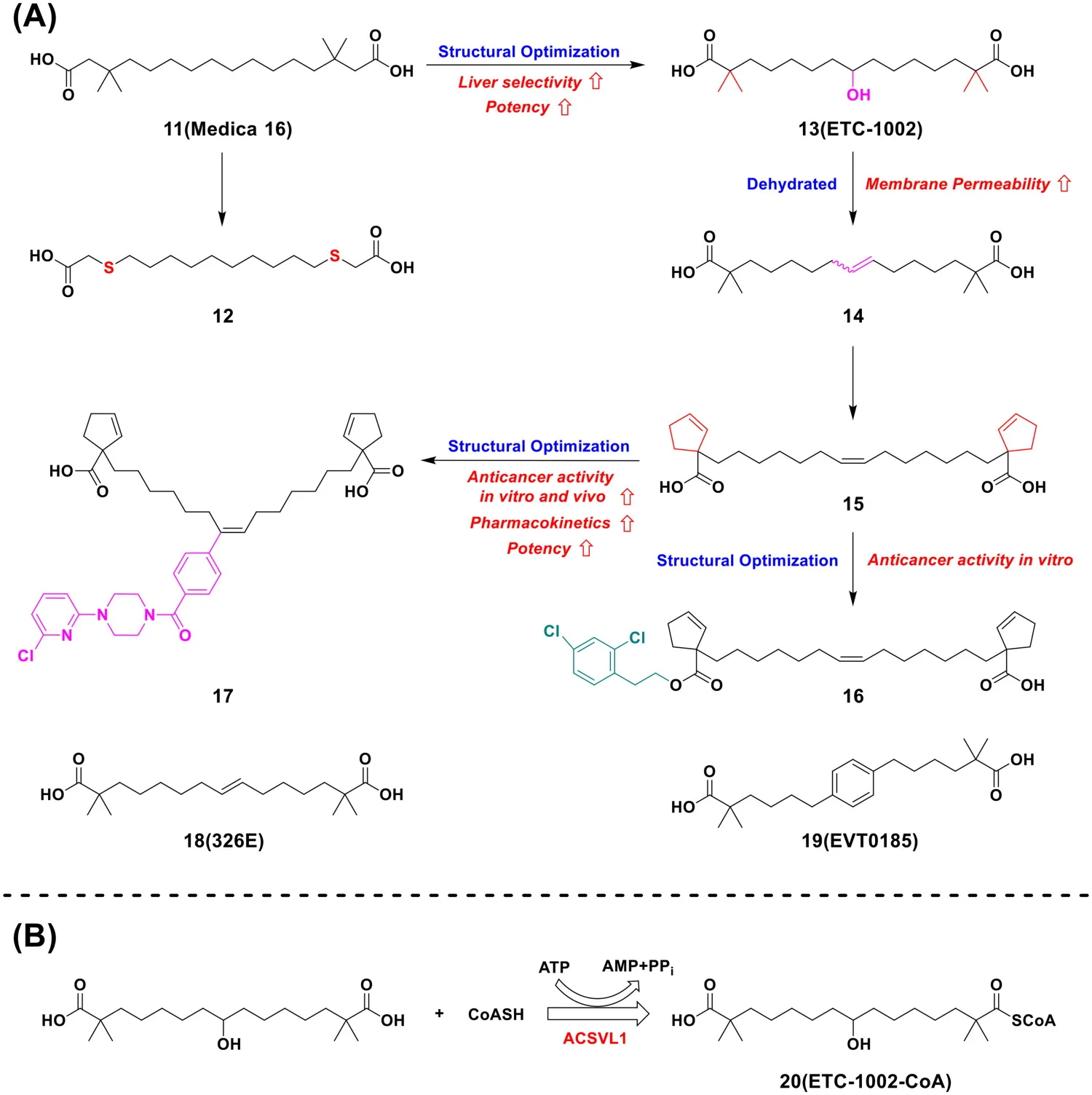
(A) The chemical structures of dicarboxylic acid analogues. (B) Activation of ETC-1002 to ETC-1002-CoA by ACSVL1. Figure was created by the author using ChemDraw software, based on references^49^^,^^112^^ ,^^115–118^.

**Figure 5.**
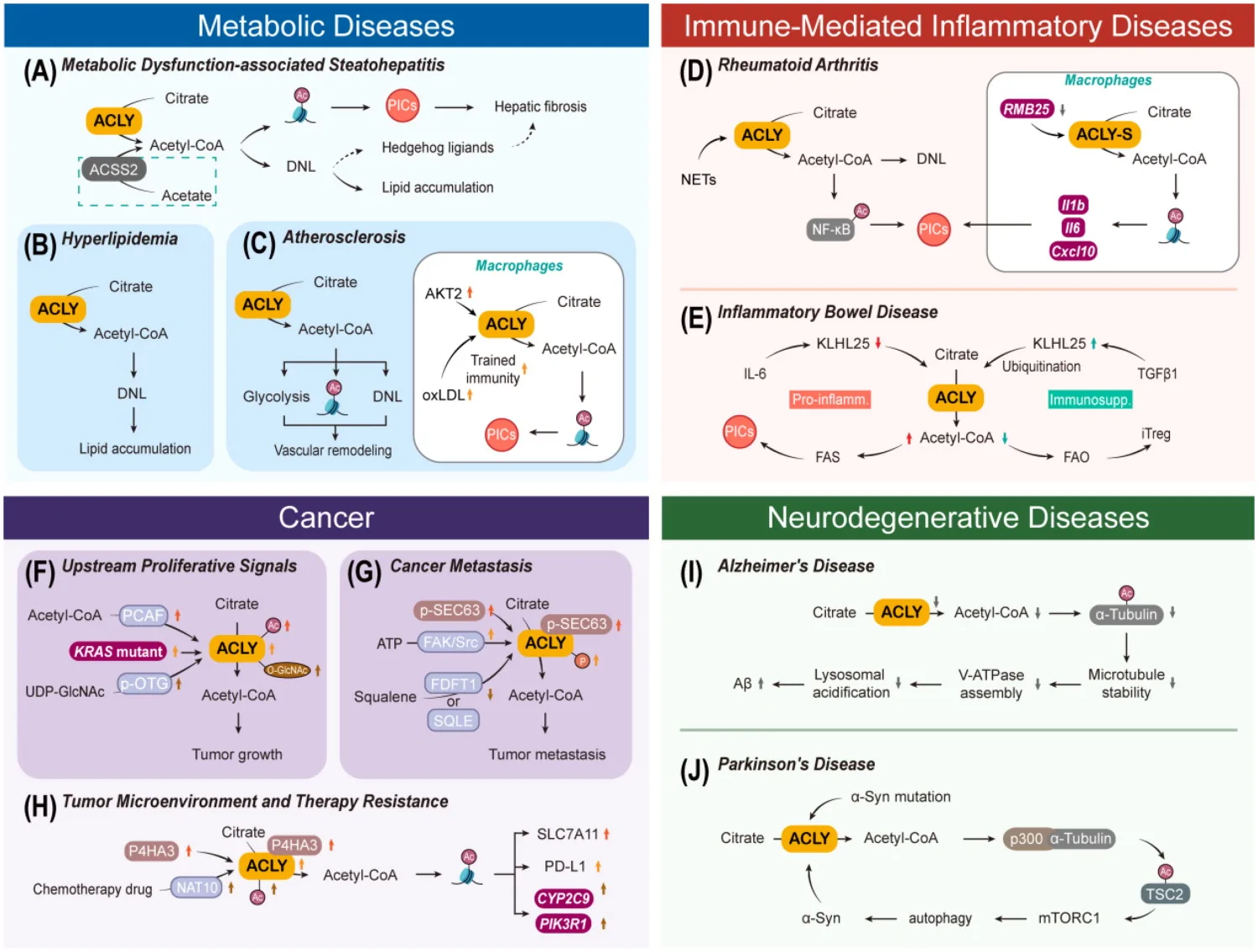
ACLY bridges metabolism and epigenetics to drive disease progression. (A) In MAFLD/MASH, ACLY drives disease progression through a shift from metabolic substrate supply in early steatosis to epigenetic reprogramming in advanced fibrosis and inflammation. (B) In hyperlipidaemia, ACLY drives excessive fatty acid and cholesterol synthesis by providing acetyl-CoA as the carbon source for hepatic DNL. (C) In atherosclerosis, ACLY drives disease progression at both systemic and local levels: it regulates hepatic lipogenesis to control plasma cholesterol levels while directly promoting vascular smooth muscle cell proliferation and macrophage epigenetic reprogramming. (D) In RA, ACLY drives disease progression through metabolic-epigenetic-inflammatory crosstalk, triggered exogenously by NETs and endogenously by *RBM25* downregulation-mediated Acly-S splicing variant. (E) In IBD, ACLY serves as a metabolic switch controlling Th17/iTreg differentiation via FAS/FAO balance in CD4^+^ T cells. (F) In tumours, upstream proliferative signals directly regulate ACLY expression and activity, while post-translational modifications further enhance its stability, positioning ACLY as a key node linking driver mutations to metabolic-epigenetic reprogramming. (G) During cancer metastasis, ACLY supports cancer cell survival and proliferation under hostile conditions by enhancing its activity via phosphorylation or increasing its stability, driving metabolic reprogramming in the metastatic niche. (H) ACLY extends its functions beyond cell-autonomous metabolism to the tumour microenvironment, mediating ferroptosis resistance, immune evasion, and chemoresistance by stabilising ACLY protein and driving H3K27ac-dependent upregulation of SLC7A11 and PD-L1. (I) In AD, ACLY loss reduces α-tubulin acetylation and V-ATPase assembly, blocking autophagy and promoting Aβ deposition. (J) In PD, α-synuclein mutations hyperactivate ACLY, leading to p300 retention and mTORC1-mediated autophagy blockade, impairing α-synuclein aggregates clearance. Figure was created by the author using Figdraw software, based on references^11^^,^^13^^,^^17^^,^^42^^,^^48^^,^^52–57^^,^^60–62^^,^^68–70^^,^^79–83^^,^^90–98^^,^^102^.

**Figure 6.**
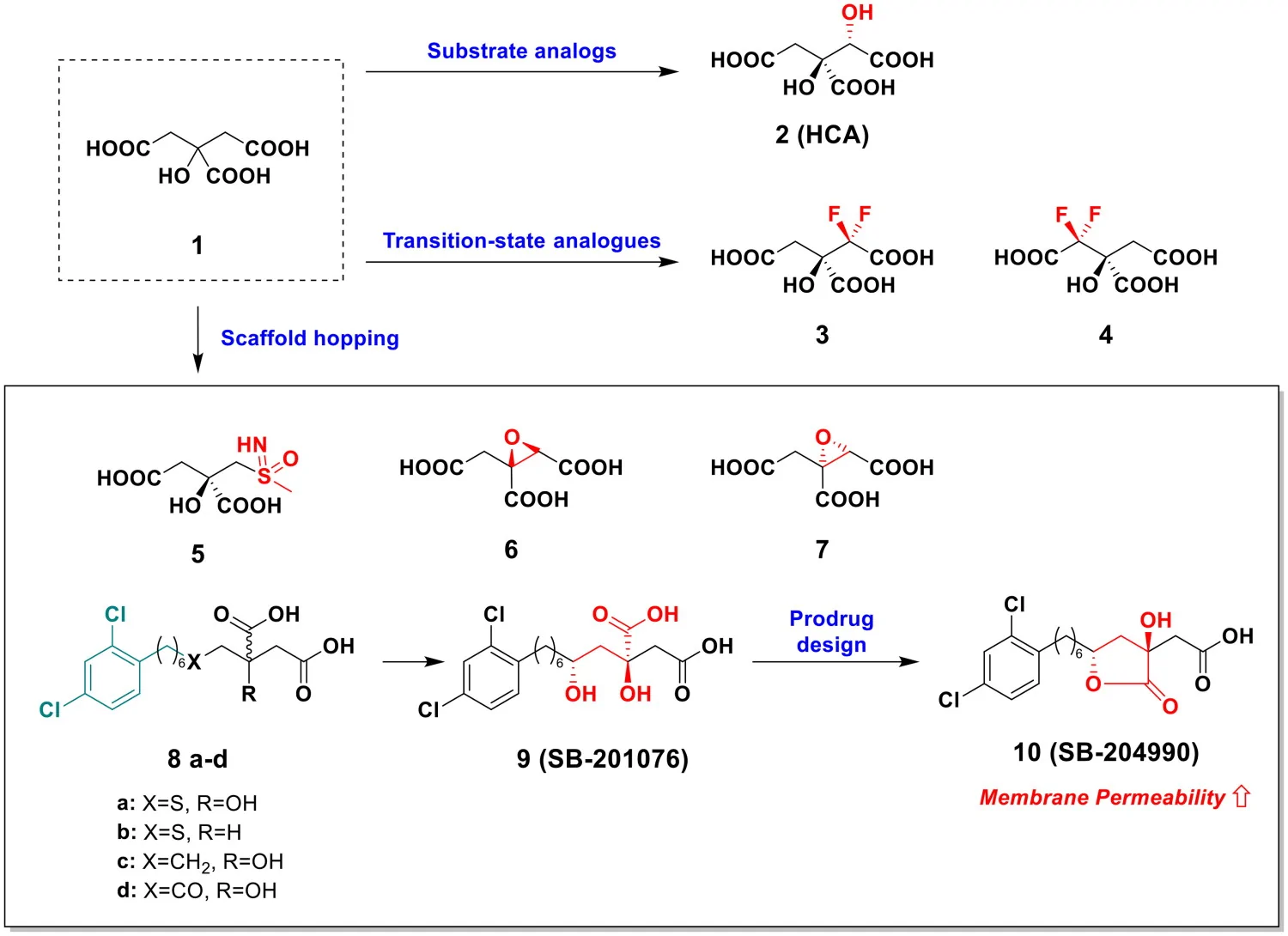
The chemical structures of citrate analogues. Figure was created by the author using ChemDraw software, based on references^105^^–^^110^.

#### Hyperlipidaemia

Hyperlipidaemia is one of the most common lipid metabolism disorders in clinical practice^58^, with its core pathological drivers considered to be excessive activation of hepatic DNL and imbalance of lipoprotein metabolism^59^. ACLY provides the carbon source required for DNL in hyperlipidaemia by catalysing the production of acetyl-CoA, contributing to the excessive generation of fatty acids and cholesterol^60^. Genetic evidence suggests that ACLY is involved in maintaining lipid homeostasis. Although *Acly* heterozygous knockout mice can maintain normal plasma lipid levels, subsequent studies have shown that liver-specific ACLY deficiency or pharmacological inhibition can reduce plasma cholesterol levels, while ACLY overexpression or enhanced activity promotes lipid synthesis (Figure 5B)^61^^,^^62^. The first approved ACLY inhibitor, bempedoic acid (compound **13**, ETC-1002, Figure 4A), has been shown to demonstrate a significant LDL-C lowering effect in a Phase III clinical trial targeting patients with hypercholesterolaemia who are intolerant to statins or exhibit inadequate lipid control after statin therapy^63^. The key CLEAR Outcomes trial (NCT02993406; *n* = 13,970) showed that compound **13** treatment significantly reduced LDL-C levels (by 21.1% versus placebo) after 6 months in statin-intolerant patients with hypercholesterolemia^63^. In Phase III trials such as CLEAR Serenity (NCT02988115) and CLEAR Tranquillity (NCT03001076), compound **13** as monotherapy or in combination with ezetimibe consistently demonstrated clear LDL-C reductions (21.4% and 28.5% versus placebo, respectively)^64^^,^^65^. Mechanistic studies have revealed that compound **13** not only inhibits the cytosolic catalytic activity and nuclear epigenetic function of ACLY, but also directly binds and activates PPARα to promote fatty acid β-oxidation^66^. This dual synergistic mechanism of “synthesis inhibition and catabolism promotion” may circumvents the compensatory risk associated with sole ACLY inhibition, thereby offering a promising multi-target strategy for the treatment of hyperlipidaemia.

#### Atherosclerosis

Systemic dyslipidemia secondary to hyperlipidaemia underlies the pathophysiology of atherosclerosis^58^, and ACLY serves as an important molecule linking this metabolic abnormality to vascular pathology. ACLY not only modulates plasma cholesterol levels by regulating hepatic lipid synthesis but also contributes to the local metabolism-inflammation-remodelling process of the vascular wall, potentially driving disease progression at both systemic and local levels^48^. In vascular smooth muscle cells (VSMCs), ACLY promotes abnormal proliferation, migration, and anti-apoptotic phenotype by promoting glycolysis (i.e., the Warburg effect), lipid synthesis, and general control non-depressible 5 (GCN5)-dependent histone acetylation, representing a key step in atherosclerotic plaque progression^48^. In a mouse femoral artery guidewire injury model, 2-week treatment with the ACLY inhibitor compound **13** significantly inhibited VSMC proliferation and dedifferentiation, thereby reducing neointimal formation in the injured artery and attenuating vascular remodeling^67^. Recent studies have shown that oxidised phospholipids (oxPAPC) enriched in atherosclerotic plaques impair the immunoregulatory function of regulatory T cells (Tregs)^68^, and that ACLY is a critical metabolic switch through which Tregs respond to this oxidised lipid signal and acquire a pro-inflammatory phenotype. ACLY also plays a role in macrophage epigenetic reprogramming. Inhibition of the AKT2 pathway reduces ACLY activation and H3K27ac acetylation, thereby suppressing inflammatory gene expression^69^; whereas cholesterol accumulation induced by oxidised low-density lipoprotein (oxLDL) reshapes histone acetylation in macrophages, forming sustained “pro-inflammatory memory” (Figure 5C)^70^. The multiple pathogenic roles of ACLY in atherosclerosis align with the cardiovascular benefits observed with compound **13** in the CLEAR Outcomes trial (NCT02993406; *n* = 13,970). This trial showed that in statin-intolerant patients with hypercholesterolaemia, compound **13** reduced the risk of major adverse cardiovascular events (MACE) by 13% (HR 0.87, 95% CI 0.79–0.96, *P* = 0.004)^63^; in the obese subgroup (BMI ≥ 30 kg/m^2^), the risk reduction reached 23%^71^. Furthermore, compound **13** significantly lowered high-sensitivity C-reactive protein (hs-CRP) levels, suggesting an anti-inflammatory effect independent of its lipid-lowering action, consistent with the aforementioned mechanistic findings of ACLY in regulating local vascular wall inflammation. Given its involvement in multiple pathological processes, ACLY is a potential target for treating metabolic and inflammatory complications associated with obesity.

### Immune-mediated inflammatory diseases

Immune-mediated inflammatory diseases (IMIDs) are a disease spectrum that encompasses classical autoimmune diseases such as rheumatoid arthritis (RA) and immune-mediated chronic inflammatory diseases such as inflammatory bowel disease (IBD)^72^^,^^73^. Obesity disrupts immune tolerance by inducing chronic low-grade inflammation, contributing to a favourable microenvironment permissive for the occurrence of IMIDs^74–76^. However, genome-wide association analysis (GWAS) shows that the strength and direction of the association between obesity and autoimmune diseases vary depending on the disease type. Obesity is positively correlated with RA, while it shows no or negative correlation with other IMIDs such as multiple sclerosis (MS) and systemic sclerosis (SSc)^77^. This differential association suggests that ACLY, as an important node of the metabolic-epigenetic network, plays a relevant role in macrophage pro-inflammatory polarisation and Treg/T helper 17 (Th17) differentiation imbalance by regulating fatty acid synthesis in immune cells and histone acetylation (regulating inflammatory gene transcription) as well as non-histone acetylation (regulating signalling protein activity and stability)^41^^,^^78^^,^^79^. In recent years, preclinical studies and bioinformatics analyses have confirmed that ACLY is abnormally activated in immune cells of IMIDs such as RA^80^, IBD^9^, thus positioning it as a potential therapeutic target. This section discusses the pathological associations and interventional evidence of ACLY in RA and IBD as representative diseases.

#### Rheumatoid arthritis

The core pathological features of RA include synovial hyperplasia, chronic inflammation, and progressive joint destruction^81^. In this process, ACLY plays an important driving role through a metabolic-epigenetic-inflammatory triple mechanism, spanning from exogenous triggering to endogenous structural dysregulation. Elevated neutrophil extracellular traps (NETs) in the synovial microenvironment can directly activate ACLY, which then triggers DNL, thereby contributing to the proliferation, migration, and invasive capacity of fibroblast-like synoviocytes (FLSs)^82^. Concurrently, ACLY also promotes the non-histone acetylation of the NF-κB p65 subunit by generating acetyl-CoA, thereby facilitating its nuclear translocation and DNA-binding ability, activating the NF-κB signalling pathway and contributing to the transcription of pro-inflammatory factors such as IL-1β and TNF-α, ultimately forming a self-reinforcing metabolic-inflammatory positive feedback loop^80^. A more fundamental mechanism lies in the structural dysregulation of ACLY in RA patient cells. Transcriptomic and functional studies have shown that downregulation of the RNA-binding motif protein 25 (RBM25) in RA patients leads to skipping of exon 14 of the Acly gene, producing a constitutively active short isoform, Acly-S. This isoform promotes glycolysis and acetyl-CoA production in macrophages, upregulates pro-inflammatory gene expression via histone H3K9ac/H3K27ac modifications, and is associated with macrophage hyperactivation (Figure 5D)^83^. In mice with macrophage-specific RBM25 deficiency, this splicing abnormality induces spontaneous arthritis, providing key in vivo functional validation of the above mechanism. Importantly, administration of the ACLY inhibitor BMS-303141 (compound **21**, Figure 7A) significantly alleviates this pathological phenotype, suggesting the therapeutic potential of targeting ACLY and its splicing regulation in RA^83^.

**Figure 7.**
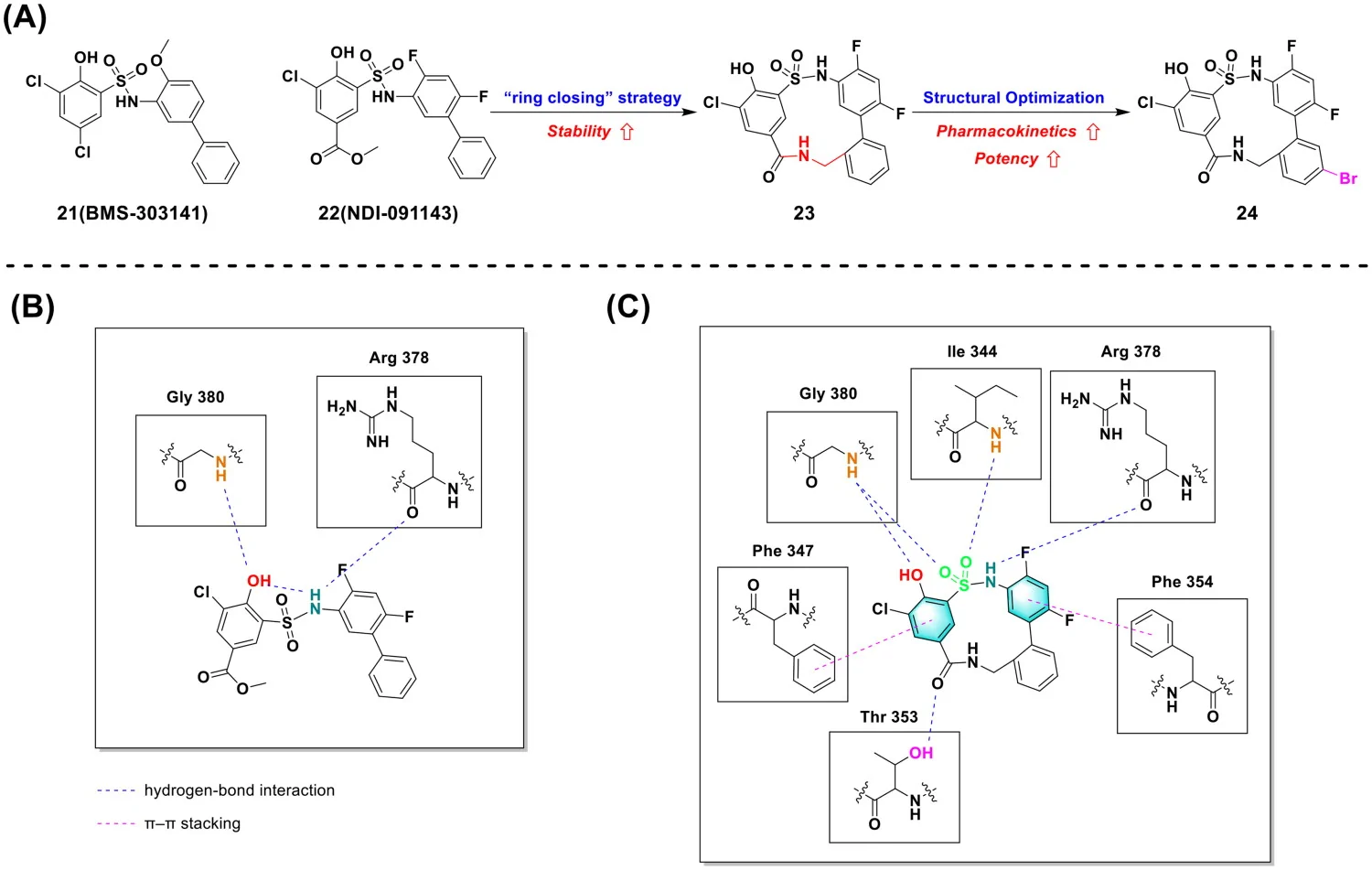
(A) The chemical structures of sulphonamide analogues (21–24). (B) Ligand-interaction diagram of NDI-091143 in the ACLY allosteric domain. (C) Ligand-interaction diagram of compound 24 in the allosteric site of ACLY. Figure was created by the author using ChemDraw software, based on references^29^^,^^119^^,^^121^^,^^122^.

#### Inflammatory bowel disease

IBD is characterised by an imbalance in CD4^+^ T cell subset differentiation as its core pathogenesis^84^^,^^85^. ACLY plays an important role in this T cell differentiation imbalance by catalysing acetyl-CoA production and regulating immune metabolism^9^. In CD4^+^ T cells, ACLY regulates its own protein stability through Kelch-like protein 25 (KLHL25)-mediated ubiquitination, thereby determining acetyl-CoA flux and the fatty acid synthesis (FAS)/fatty acid oxidation (FAO) metabolic switch, which serves as the core metabolic switch governing CD4^+^ T cell differentiation into T helper 17 (Th17) or inducible regulatory T cells (iTreg) (Figure 5E)^79^. Under steady-state conditions, ACLY is highly expressed in normal colonic tissue; however, in dextran sulphate sodium (DSS)-induced experimental colitis mouse models and IBD patients, ACLY expression is significantly downregulated in CD4^+^ T cells (and CD8^+^ T cells) infiltrating the inflamed intestine, and ACLY-deficient CD4^+^ T cells exhibit reduced pro-inflammatory capacity^9^. The metabolic regulatory function of ACLY may not be limited to immune cells. In intestinal epithelial cells, ACLY is also expressed and involved in acetyl‑CoA metabolism. Downregulation of its function may lead to impaired barrier integrity, thereby exacerbating abnormal activation of submucosal immune cells. A 2025 study found that the expression of ACSS2, another key enzyme in the acetyl‑CoA biosynthesis pathway, was significantly downregulated in the intestinal epithelium of IBD patients. Its deficiency can directly impair barrier function and exacerbate colitis^86^. This suggests that the damage to the epithelial barrier caused by acetyl‑CoA metabolic disorder and the pro‑inflammatory effect of T cells are interdependent, coupled through a feed-forward loop of “epithelial barrier disruption–immune cell activation,” collectively driving the chronic inflammatory process of IBD^86^. Collectively, these findings indicate that targeting ACLY can provide new strategies for modulating autoimmune pathology by interfering with the metabolic-epigenetic coupling of pathogenic immune cells.

### Cancer

The occurrence and malignant progression of tumours are highly dependent on sustained proliferative signals, metabolic reprogramming, and the acquisition of metastatic capacity^26^^,^^87^. Cancer cells typically exhibit metabolic hallmarks such as the Warburg effect and upregulated DNL to meet their demands for sustained proliferation, invasion, and metastasis. The acetyl-CoA generated by ACLY serves not only as a substrate for lipid synthesis and a donor for histone acetylation, but also drives the acetylation of various non-histone proteins^20^^,^^88^^,^^89^, thereby providing dual metabolic and epigenetic support for tumour growth across carcinogenesis, progression, and metastasis^18^^,^^90^.

Upstream proliferative signals regulate ACLY expression and activity, positioning ACLY as an important node linking oncogenic mutations to metabolic-epigenetic reprogramming. In pancreatic cancer, KRAS gene mutations drive ACLY overexpression and activation, leading to acetyl-CoA metabolic reprogramming that is already initiated in the precancerous stage^90^. Post-translational modifications of ACLY itself further fine-tune its function. Studies have reported that in lung cancer, p300/CBP-associated factor (PCAF) mediates acetylation of ACLY at residues K540, K546, and K554, which blocks its ubiquitination and proteasomal degradation, thereby enhancing ACLY stability to support tumour growth; conversely, Sirtuin 2 (SIRT2)-mediated deacetylation reduces ACLY stability, contributing to decreased lipid synthesis and cell proliferation^91^. In glioblastoma, Phosphatidylinositol 3-kinase (PI3K)β acts as a protein kinase to phosphorylate O-GlcNAc transferase (OGT), which in turn mediates O-GlcNAcylation of ACLY (T639/S667). Activated ACLY promotes acetyl-CoA production while simultaneously promoting fatty acid synthesis and histone H3 acetylation, driving tumour growth (Figure 5F)^92^^,^^93^. In addition, acetyl-CoA derived from ACLY can directly drive acetylation of non-histone proteins, achieving rapid regulation of signalling protein activity and stability. In head and neck squamous cell carcinoma, ACLY promotes NF-κB p65 acetylation to modulate its transcriptional activity^89^; in various tumours, ACLY maintains acetylation of the K168 site of ferroptosis suppressor protein 1 (FSP1) by providing acetyl-CoA, preventing it from being degraded by the proteasome through the K29-linked ubiquitin, thereby conferring ferroptosis resistance^20^.

During tumour metastasis, ACLY supports the survival and dissemination of cancer cells in stressful microenvironments through various mechanisms. In oesophageal squamous cell carcinoma, focal adhesion kinase (FAK)/Src kinase phosphorylates ACLY at Tyr542/652, promoting its activity and supporting metabolic reprogramming to promote the survival and proliferation of tumour cells in metastatic lymph nodes^94^. In hepatocellular carcinoma, endoplasmic reticulum stress activates inositol-requiring enzyme 1α (IRE1α) kinase, which phosphorylates SEC63, thereby stabilising ACLY. ACLY in turn not only synergistically upregulates Snail1 expression to promote cancer cell metastasis, but also increases acetyl-CoA supply to support ER homeostasis and support tumour cell survival under stress conditions (Figure 5G)^95^. In colorectal cancer, loss of the cholesterol synthesis enzymes farnesyl-diphosphate farnesyltransferase 1 (FDFT1) or squalene epoxidase (SQLE) enhances tumour metastasis and stemness, whereas SB-204990 (compound **10**, Figure 6) blocks this process, suggesting that ACLY is an important downstream node in this pro-metastatic pathway^96^.

The role of ACLY extends beyond cell-autonomous mechanisms to the tumour microenvironment, where it participates in immune evasion, chemoresistance, and immune remodelling. In cervical cancer, prolyl 4-hydroxylase subunit alpha 3 (P4HA3) stabilises ACLY protein by competitively inhibiting the interaction between the E3 ubiquitin ligase UBR4 and ACLY; ACLY-derived acetyl-CoA promotes transcription of solute carrier family 7 member 11 (SLC7A11) by enhancing H3K27ac levels in its promoter region, ultimately conferring ferroptosis resistance and driving lymphatic metastasis of cervical cancer^97^. In melanoma, ACLY-generated acetyl-CoA promotes PD-L1 transcription and expression via P300-mediated histone H3K27ac acetylation, mediating tumour immune evasion^17^. Clinical samples have confirmed that this mechanism is associated with adverse responses to immunotherapy, while targeting ACLY restores cytotoxic T cell function and enhances immunotherapy efficacy. In hepatocellular carcinoma, chemotherapeutic agents induce the acetyltransferase N-acetyltransferase 10 (NAT10) to acetylate ACLY at K468, promoting ACLY accumulation in the nucleus. By increasing the nuclear acetyl-CoA pool and H3K27ac levels, this modification activates the transcription of chemoresistance genes such as cytochrome P450 2C9 (CYP2C9) and phosphoinositide 3-kinase regulatory subunit 1 (PIK3R1), thereby driving chemoresistance (Figure 5H)^98^. The latest research further reveals that in MASH-driven hepatocellular carcinoma, oral administration of the ACLY inhibitor compound **19** promotes B cell infiltration and tertiary lymphoid structure formation by upregulating C-X-C motif chemokine ligand 13 (CXCL13), modulating the tumour immune microenvironment and enhancing immunotherapy efficacy^49^.

### Neurodegenerative diseases

In metabolic diseases, IMIDs and cancer, ACLY dysregulation is commonly manifested as overactivation, providing lipid precursors and epigenetic substrates for cell proliferation^99^. However, in the nervous system, neurons are terminally differentiated cells whose pathological features are not proliferation but rather proteostasis imbalance, energy metabolism dysfunction, and synaptic loss^100^^,^^101^. Recent studies have suggested a context-dependent role of ACLY in regulating protein homeostasis in neurodegenerative diseases.

In Alzheimer’s disease (AD), ACLY expression and activity are critical for maintaining α-tubulin acetylation and V-ATPase assembly^11^. Reduced neuronal ACLY levels in AD patients and model mice lead to decreased α-tubulin acetylation and impaired microtubule stability, which in turn hinder V-ATPase assembly and lysosomal acidification, ultimately causing autophagy flux impairment and enhanced Aβ deposition (Figure 5I). Conversely, in Parkinson’s disease (PD), mutations in the α-synuclein gene can hyperactivate ACLY, resulting in cytoplasmic accumulation of acetyl-CoA and cytoplasmic retention with aberrant activation of p300 acetyltransferase, thereby blocking autophagy flux via Mammalian target of rapamycin complex 1 (mTORC1) hyperactivation^102^. In the context of PD pathology, the specific ACLY inhibitors hydroxycitrate (compound **2**, HCA, Figure 6) and compound **10** effectively reduce cytoplasmic acetyl-CoA levels, promote p300 nuclear translocation, restore autophagy function, and enhance clearance of α-synuclein aggregates, as well as ameliorate pathological phenotypes such as motor deficits and neuronal death in human induced pluripotent stem cell (iPSC)-derived neurons, brain organoids, zebrafish, and mouse models (Figure 5J). Therefore, ACLY represents a potential target for neuroprotection or disease modification^102^.

## Development and molecular design of ACLY inhibitors

Previous sections have outlined the involvement of ACLY in the pathogenesis of various diseases, underscoring its considerable potential as a therapeutic target. Although pharmacological inhibition of ACLY has demonstrated benefits across multiple disease models, and some inhibitors have advanced to clinical trials, most remain in preclinical development. Existing reviews have summarised the structural optimisation and structure-activity relationships (SAR) of ACLY inhibitors, yet they often focus on specific disease contexts or particular chemotypes. Building on this foundation, this review provides a systematic analysis of the structural optimisation, structure-activity relationships, and design strategies of ACLY inhibitors based on diverse core scaffolds. It also surveys both synthetic and natural ACLY inhibitors (Tables 1 and 2), summarising the current landscape to inform the development of next-generation inhibitors with improved selectivity and tissue specificity.

**Table 1.** Summary table for natural ACLY inhibitors.

Compound	Biological activities	Used methods	Clinical trial ID or refs
Clinical trial inhibitors			
HCA **(2)**	K_i_ = 8 μM (ACLY from rat liver)	enzymatic assay (NADH-coupled spectrophotometry)	^105^
	Obesity and type 2 diabetes		NCT00699413 (terminated)
	Obesity and Oxidative Stress		NCT01238887 (terminated)
	urine chemistries		NCT03348228 (not applicable)
Preclinical stage inhibitors			
Compounds **(29)**	IC_50_ = 283 nM (ACLY from rat liver)	enzymatic assay (NADH-coupled spectrophotometry)	^127^
	K_i_ < 100 nM (ACLY from rat liver)	enzymatic assay (NADH-coupled spectrophotometry)	
Purpurone **(35)**	IC_50_ = 7 μM (Enzyme source not specified)	enzymatic assay (NADH-coupled spectrophotometry)	^132^
Compounds **36a–b**	K_i_ = 4.0–4.2 μM (ACLY from rat liver)	enzymatic assay (Hydroxamate method)	^133^
Radicicol **(37)**	K_i_ = 13 μM (ACLY from rat liver, vs citrate)	enzymatic assay (NADH-coupled spectrophotometry)	^134^
	K_i_ = 7 μM (ACLY from rat liver, vs ATP)	enzymatic assay (NADH-coupled spectrophotometry)	
Cucurbitacin B **(38)**	IC_50_ ≈ 0.3 μM (antiproliferative activity in prostate cancer cells)	Cell-based assay	^135^
	inhibited cancer cell growth	in vitro and in vivo studies	
Compounds **39**	IC_50_ = 0.44 μM (recombinant human ACLY)	enzymatic assay (ADP-Glo)	^136^
ICC **(40)**	IC_50_ = 0.14 μM (recombinant human ACLY)	enzymatic assay (ADP-Glo)	^129^
Styraxlignolide D **(41)**	IC_50_ = 7.62 μM (Enzyme source not specified)	enzymatic assay	^137^
ISOGK **(42)**	IC_50_ = 5.46 μM (recombinant human ACLY)	enzymatic assay (ADP-Glo)	^138^
	mitigates hypercholesterolaemia and atherosclerosis	in vitro and in vivo studies	
PB2 **(43)**	IC_50_ = 2.73 μM (recombinant human ACLY)	enzymatic assay (ADP-Glo)	^139^
	reduced lipid accumulation	in vitro studies	
Brevifolin D**(44)**	IC_50_ = 9.6 μM (ACLY)	enzymatic assay (ADP-Glo)	^140^
	IC_50_ = 11.0 μM (ACC1)	enzymatic assay (ADP-Glo)	

**Table 2.** Summary table for synthetic ACLY inhibitors.

Compound	Biological activities	Used methods	Clinical trial ID or refs
Clinical trial inhibitors			
Bempedoic acid **(13**) and ETC-1002-CoA (**20**)	Active metabolite (**20**): K_i_ = 2 μM (recombinant human ACLY)	Radiometric assay ([^14^C]-citrate)	^115^
	primary hypercholesterolaemia and established atherosclerotic cardiovascular disease		approved
326E **(18)**	Active metabolite: IC_50_ = 5.31 μM (recombinant human ACLY)	enzymatic assay (ADP-Glo)	^118^
	Primary hypercholesteremia		CTR20240756 (Phase II)
	Mixed hyperlipidaemia		ChiCTR2600115928 (Phase IIa)
	MASH		NCT06491576 (Phase Ib/IIa)
Preclinical stage inhibitors			
Compound **3**	K_i_ = 0.7 μM (ACLY from rat liver)	enzymatic assay (NADH-coupled spectrophotometry)	^106^
Compound **4**	K_i_ = 3.2 μM (ACLY from rat liver)	enzymatic assay (NADH-coupled spectrophotometry)	^106^
Compound **5**	K_i_ = 250 μM (ACLY from rat liver)	enzymatic assay (NADH-coupled spectrophotometry)	^107^
Compound **6**	K_i_ = 18 μM (ACLY from rat liver)	enzymatic assay (NADH-coupled spectrophotometry)	^108^
Compound **7**	K_i_ = 400 μM (ACLY from rat liver)	enzymatic assay (NADH-coupled spectrophotometry)	^108^
Compounds **8a–d**	K_i_ = 1.2–3.3 μM (ACLY from rat liver)	enzymatic assay (NADH-coupled spectrophotometry)	^109^
SB-201076 **(9)**	K_i_ = 1 μM (rat and human ACLY)	enzymatic assay (NADH-coupled spectrophotometry)	^110^
SB-204990 **(10)**	reduction of cholesterol and fatty acid synthesis	in vitro and in vivo studies	^111^
MEDICA 16 **(11)**	K_i_ = 16 μM (ACLY from rat liver)	enzymatic assay (NADH-coupled spectrophotometry)	^112^
Compound **12**	reduction of plasma triglycerides and cholesterol	in vivo assays	^113^
Compound **14**	IC_50_ = 55.7 μM (recombinant human ACLY)	enzymatic assay (ADP-Glo)	^116^
Compound **15**	IC_50_ = 1.5 μM (recombinant human ACLY)	enzymatic assay (ADP-Glo)	^116^
	inhibited cancer cell growth	in vitro assays	
Compound **16**	IC_50_ = 3.1 μM (recombinant human ACLY)	enzymatic assay (ADP-Glo)	^116^
	inhibited cancer cell growth	in vitro assays	
compound **17**	IC_50_ = 0.87 μM (recombinant human ACLY)	enzymatic assay (ADP-Glo)	^117^
	reduced plasma lipids and inhibited cancer cell growth	in vitro and in vivo studies	
EVT0185 **(19)**	Active metabolite: IC_50_ = 2.5 μM (recombinant human ACLY)	enzymatic assay (ADP-Glo)	^49^
	Active metabolite: IC_50_ = 45 μM (recombinant human ACSS2)	enzymatic assay (ADP-Glo)	
	inhibited MASH-HCC	in vitro and in vivo studies	
BMS-303141 **(21)**	IC_50_ = 0.13 μM (recombinant human ACLY)	enzymatic assay (NADH-coupled spectrophotometry)	^119^
	inhibits lipid synthesis, lowers plasma cholesterol and triglycerides, and reduces body weight gain	in vitro and in vivo studies	
NDI-091143 **(22)**	IC_50_ = 2.1 nM (recombinant human ACLY)	enzymatic assay (ADP-Glo)	^29^
Compound **23**	IC_50_ = 69.7 nM (recombinant human ACLY)	enzymatic assay (NADH-coupled spectrophotometry)	^121^
Compound **24**	IC_50_ = 8.3 nM (recombinant human ACLY)	enzymatic assay (NADH-coupled spectrophotometry)	^122^
	hypolipidemic	in vitro and in vivo studies	
Compounds **25a–e**	IC_50_ = 0.47–0.86 μM (recombinant human ACLY)	enzymatic assay (ADP-Glo)	^124^
Compound **27**	IC_50_ = 13.8 μM (recombinant human ACLY)	enzymatic assay (ADP-Glo)	^126^
Compound **28a–b**	IC_50_ = 4.1–11.9 μM (recombinant human ACLY)	enzymatic assay (ADP-Glo)	^126^
Compound **31**	IC_50_ = 2.9 μM (recombinant human ACLY)	enzymatic assay (ADP-Glo)	^128^
	inhibited cancer cell growth	in vitro assays	
Compound **33**–**34**	IC_50_ =22-36 nM (recombinant human ACLY)	enzymatic assay (ADP-Glo)	^131^
	ameliorated weight gain, hepatic steatosis, dyslipidemia, liver inflammation, and fibrosis	in vitro and in vivo studies	

### Citrate analogues

Hydroxycitric acid (compound 2, HCA, Figure 6), isolated from Garcinia cambogia, was the first reported ACLY inhibitor (K_i_ = 8 μM), which suppresses acetyl-CoA production by competitively inhibiting citrate binding^103–105^. Following the approach of the toxicity mechanism, Wells et al. introduced a C–F bond into citrate (compound **1**) and, following chiral resolution, obtained (3 R)-(+)-2,2-difluorocitrate (compound **3**, K_i_ = 0.7 μM), a selective ACLY inhibitor, and its (3S)-(−)-isomer (compound **4**, K_i_ = 3.2 μM), which inhibits aconitase^106^. Dolle et al. Subsequently designed and synthesised sulfoximine inhibitors (e.g., compound **5**) by mimicking CoA or its reaction intermediates, rationalised by ACLY’s bisubstrate catalytic mechanism; however, these exhibited weak binding affinity (K_i_ = 250 μM)^107^. The same group further developed epoxide derivatives (compounds **6** and **7**) aimed at irreversible inhibition via covalent modification of the active site, but these also showed limited potency (K_i_ = 18 μM and 400 μM, respectively)^108^. Drawing inspiration from statins, Gribble et al. constructed a series of compounds featuring a hydrophobic “citrate-like” head group (compounds **8a–b**; K_i_ = 1.2–3.3 μM), which, however, suffered from poor cellular permeability^109^. Through SAR optimisation and prodrug design, SB-201076 (compound **9**, K_i_ = 1 μM) and its prodrug SB-204990 (compound **10**) were developed^110^. After intracellular conversion, the active metabolite of compound **10** reduced fatty acid and cholesterol synthesis in colorectal cancer cells to 32% and 8% of control levels, respectively^111^.

### Dicarboxylic acid analogues

Early efforts primarily employed a substrate-mimetic strategy. Bar-Tana et al. reported the dicarboxylic acid ACLY inhibitor Medica 16 (compound **11**, Figure 4A), which is proposed to occupy the substrate-binding pocket by mimicking citrate. With a K_i_ of 16 μM (using citrate as substrate in rat liver), it preliminarily validated the dicarboxylic acid scaffold as a potential core for ACLY inhibition^112^. Building on this scaffold, researchers replaced the β-dimethyl group with a sulphur atom to yield compound **12**, a modification designed to modulate electronic distribution and conformational flexibility. In animal models of nephrotic hyperlipidaemia, this compound was reported to reduced plasma triglyceride and cholesterol levels^113^. This scaffold-modification strategy thereby laid the structural groundwork for the subsequent development of bempedoic acid. Esperion Therapeutics developed bempedoic acid (compound **13**, ETC-1002), which was approved by the FDA and EMA in 2020 for treating hypercholesterolemia^114^^,^^115^. Its approval was primarily based on clinical data from the CLEAR Outcomes trial demonstrating reductions in LDL-C levels and MACE risk (see Sections 2.1.2 and 2.1.3). compound **13** is a prodrug that is converted by the hepatic enzyme very long-chain acyl-CoA synthetase-1 (ACSVL1) into its active form, ETC-1002-CoA (compound **20**, K_i_ = 2 μM, Figure 4B)^115^, which directly inhibits ACLY.

To further optimise drug-like properties, Li et al. conducted detailed SAR studies. Starting from the long-chain alkene diacid scaffold compound **14** (IC_50_ = 55.7 μM), iterative substituent screening led to the potent ACLY inhibitor compound **15** (IC_50_ = 1.5 μM), establishing a reference SAR for this scaffold^116^. Structural analysis suggested the presence of an underutilised hydrophobic cavity within the ACLY binding pocket. Introducing hydrophobic groups complementary to this cavity was hypothesised to enhance activity. Based on this, the team introduced the 2,4-dichlorophenethyl moiety into the scaffold of compound 15 through an esterification-based derivatization strategy, yielding compound **16** (IC_50_ = 3.1 μM)^116^. Although the enzyme inhibitory potency slightly decreased, this modification demonstrated the potential utility of the hydrophobic cavity and, as an ester derivative, also enhanced both antiproliferative activity and membrane permeability.

Using compound **15** as a starting point, the team performed precise binding mode analysis via molecular docking, which revealed an adjacent subpocket near the CoA-binding site. Based on this structural insight, they employed a fragment-based drug design strategy. Through iterative SAR optimisation, a tridentate scaffold was designed, yielding compound **17** (IC_50_ = 0.87 μM)^117^. This compound exhibits substantial ACLY inhibitory activity and acceptable pharmacokinetic properties, with its in vivo efficacy in reducing tumour burden and liver fibrosis demonstrated in a mouse model of hepatocellular carcinoma (HCC)^117^. Derived from this series, 326E (compound **18**; active metabolite IC_50_ = 5.31 μM) entered clinical trials in 2022 for hypercholesterolaemia, with its preclinical efficacy also demonstrated in MASH models (see Sections 2.1.1)^118^. Additionally, Gregory R. Steinberg’s team reported the ACLY/ACSS2 dual-target inhibitor EVT0185 (compound **19**; active metabolite ACLY IC_50_ = 2.5 μM, ACSS2 IC_50_ = 45 μM)^49^. This compound is converted into an active CoA thioester by SLC27A2 in the liver and directly interacts with the CoA-binding pocket of ACLY. Compound 19 alleviates steatosis, hepatic stellate cell activation, and fibrosis in MASH mouse models, and this dual-targeting strategy has been evaluated in vivo in MASH and MASH-HCC models^54^.

### Sulphonamide analogues

In 2007, Bristol-Myers Squibb identified a series of 2-hydroxy-N-phenylsulfonamide compounds via high-throughput screening. A potent analogue was BMS-303141 (compound **21**, IC_50_ = 0.13 μM, Figure 7A)^119^. It effectively inhibited lipid synthesis in HepG2 cells and showed no cytotoxicity in normal human hepatocytes^120^.In 2019, Wei et al. reported a congener, NDI-091143 (compound **22**, IC_50_ = 2.1 nM), which demonstrated enhanced potency compared to BMS-303141, albeit with a relatively short half-life in human liver microsomes (T_1/2_ = 3.36 min)^29^. The cocrystal structure showed that NDI-091143 binds to an allosteric site of ACLY in a U-shaped folded conformation. Within this conformation, the phenolic hydroxyl forms a hydrogen bond with the backbone amide of Gly380, and the sulphonamide nitrogen donates a hydrogen bond to the carbonyl of Arg378. An intramolecular hydrogen bond between the phenolic hydroxyl and the sulphonamide groups further stabilises this U-shaped geometry, leading to allosteric inhibition (Figure 7B)^29^.

In 2022, Zang et al. applied a cyclisation strategy to conformationally constrain NDI-091143, yielding macrocyclic compound **23**. Molecular docking suggested that compound 23 maintains key interactions: its phenolic hydroxyl with Gly380, sulphonamide nitrogen with Arg378, and a sulphonamide oxygen with Ile344. Additionally, its aromatic rings engage in π–π stacking with Phe347 and Phe354, collectively stabilising the ligand–protein complex (Figure 7C). Although the potency of compound **23** decreased (IC_50_ = 69.7 nM), its metabolic stability in human liver microsomes was substantially improved (T_1/2_ = 531 min)^121^. Docking also indicated that the biphenyl moiety of compound **23** occupies a hydrophobic subpocket comprising residues including Ile357^121^. Subsequent SAR studies confirmed the importance of the phenolic hydroxyl, as its removal led to loss of activity. Building on this, introducing a bromine atom at the biphenyl position provided compound **24**, which maintained good potency (IC_50_ = 8.3 nM) alongside improved pharmacokinetic properties^122^.

### Flavonoid analogues

Flavonoids are a class of natural products with anti-inflammatory, antioxidant, and anti-tumour properties^123^. In 2022, Wang et al. identified a series of ACLY inhibitors (IC_50_ = 0.47–0.86 μM) via high-throughput screening of 138 flavonoid derivatives. The most active compounds included herbacetin (compound **25a**, IC_50_ = 0.5 μM, Figure 8), quercetin (compound **25b**, IC_50_ = 0.86 μM), luteolin (compound **25c**, IC_50_ = 0.64 μM), scutellarin (compound **25d**, IC_50_ = 0.71 μM), and myricetin (compound **25e**, IC_50_ = 0.47 μM)^124^. SAR studies revealed that the conjugated system formed by the ortho-dihydroxyphenyl and pyranone rings is important for inhibitory activity, whereas 5-hydroxymethylation led to loss of potency. Enzyme kinetic analysis characterised compound **25a** as a non-competitive inhibitor against hACLY, with K_i_ values of 0.11 μM (against citrate) and 0.19 μM (against CoA), suggesting binding to an allosteric site. Molecular docking studies further indicated that herbacetin forms hydrogen bonds with Asn346, Thr348, Asn349, and Thr353, while its benzene ring engages in π–π stacking interactions with Phe354 (Figure 8)^124^.

**Figure 8.**
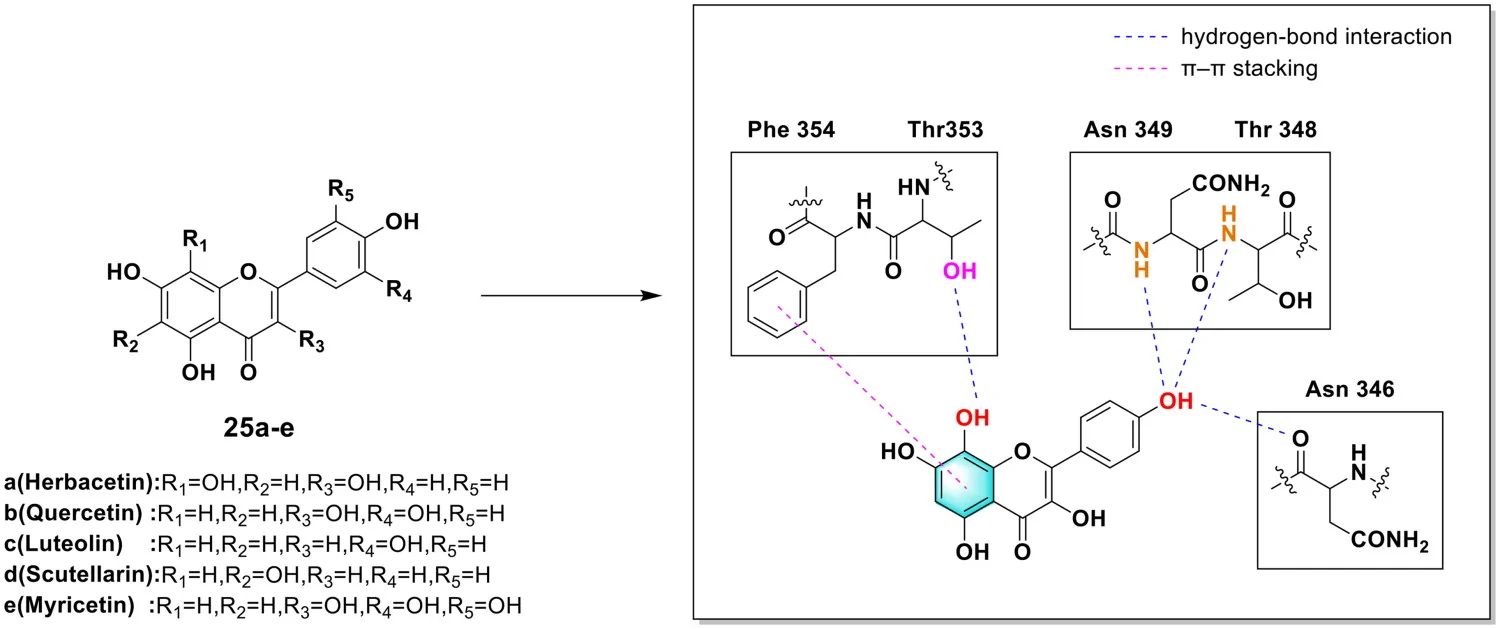
The chemical structures of flavonoid analogues (25a–e) and ligand-interaction diagram of herbacetin in the active site of ACLY. Figure was created by the author using ChemDraw software, based on references^124^.

### Furan carboxylate analogues

In 1985, Hall et al. reported that 2-furoic acid (compound **26**, Figure 9) and 3-furoic acid could inhibit ACLY activity in rodent liver, albeit at high concentrations^125^. Building on this, Jernigan et al. employed 2-furoic acid as a scaffold for virtual screening in 2017, identifying furan carboxylic acid derivatives. Among these, the 5-sulfonamidobenzofuran-3-carboxylic acid derivative compound **27** (IC_50_ = 13.8 μM) and 4-substituted-2-furoic acid analogue compound **28a** were the most potent (IC_50_ = 4.1 μM), while its homologue compound **28b** (IC_50_ = 11.9 μM) showed weaker activity. SAR analysis suggested that the R_2_ substituent of compound **28a** extends into a hydrophobic subpocket, forming additional hydrophobic or π–π stacking interactions, which explains its superior activity over compound **28b**. The study evaluated compound activity using an ADP-Glo enzymatic assay and by measuring lipid synthesis in HepG2 cells, though the determination of a cocrystal structure remains pending^126^. These findings validate the 2-furoic acid scaffold as a viable lead for ACLY inhibitors and highlight the 4-position as a site for subsequent structural optimisation through modulation of substituents.

**Figure 9.**
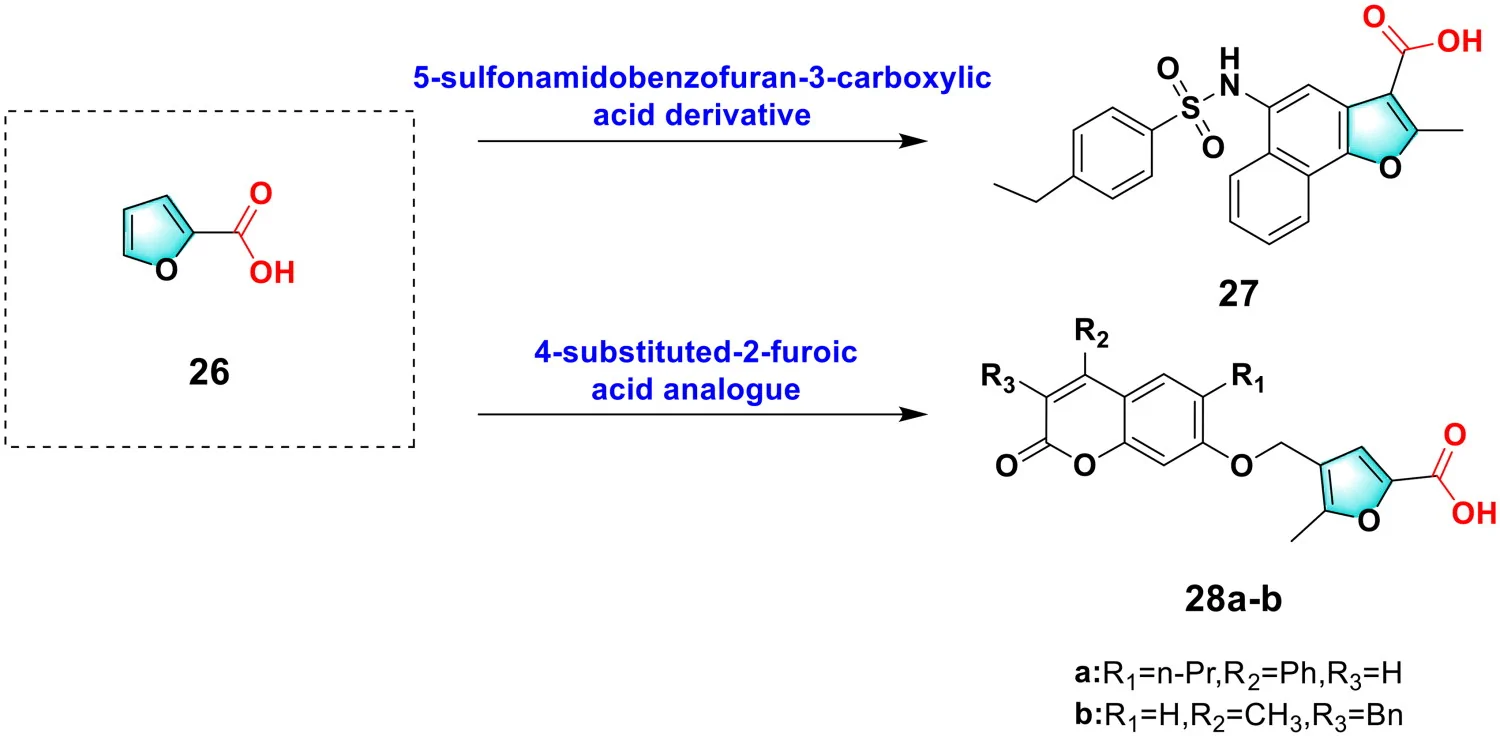
The chemical structures of furan carboxylate analogues. Figure was created by the author using ChemDraw software, based on references^125^^,^^126^.

### Anthraquinone analogues

In 1995, Oleynek et al. isolated a series of ACLY inhibitors from the fungal culture SC2193. One of the most potent among them was 2-chloro-1,3,8-trihydroxy-6-methyl-10H-anthracen-9-one (compound **29**, IC_50_ = 283 nM, K_i_ < 100 nM, Figure 10)^127^. The structurally similar natural product Emodin (compound **30**), which lacks the C-2 chlorine atom, exhibited reduced activity. However, its anthraquinone scaffold offers improved stability, which is advantageous for drug development. Koerner et al. subsequently conducted SAR studies based on the Emodin scaffold. They confirmed the critical importance of a substituent at the C-2 position and found that introducing a bromine atom at C-4 could further enhance interactions with hydrophobic residues in the allosteric site. Compound **31** (IC_50_ = 2.9 μM), which bears bromine atoms at both C-2 and C-4, was among the most potent analogues in the series. Its direct inhibition of ACLY and anti-proliferative effects were assessed using enzyme activity assays and an A549 cell model, providing experimental support for the molecular docking predictions^128^.

**Figure 10.**
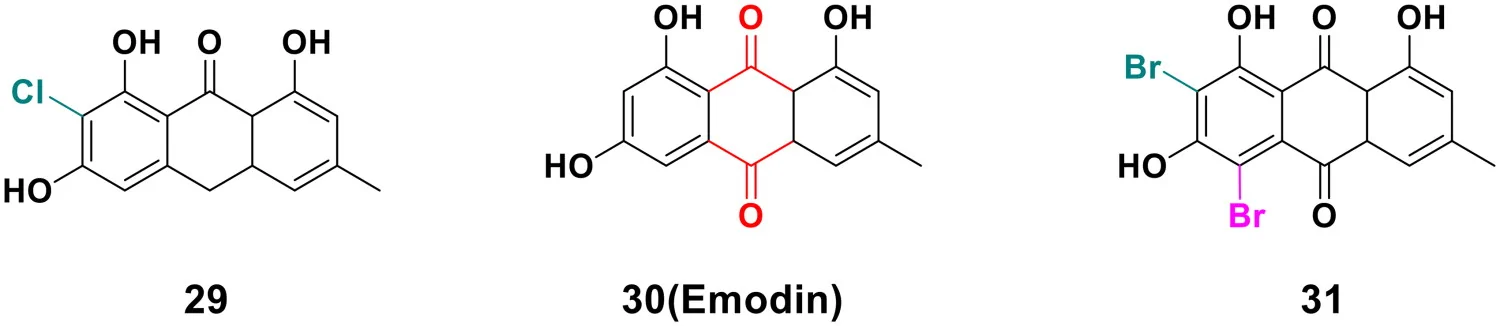
The chemical structures of anthraquinone analogues. Figure was created by the author using ChemDraw software, based on references^127^^,^^128^.

### Chalcone skeleton analogues

Based on SAR studies of various natural ACLY inhibitors^129^^,^^130^, Xu et al. designed and synthesised a series of chalcone-based compounds (compound **32**, Figure 11). Among these, derivatives featuring a five-membered ring (compound **33**) or a rigid benzofuran scaffold (compound **34**) showed potent activity (IC_50_ = 36 nM and 22 nM, respectively). Hydroxyl groups were suggested to be important for activity. Molecular docking suggested that compound **33** forms hydrogen bonds with ACLY residues including Gly341, Gly380, and Pro382, and engages in π–π stacking with Phe347. In contrast, compound **34** forms a hydrogen bond with Arg378 and participates in π–π stacking interactions with Tyr307 and Phe354; it may also mediate indirect interactions via water molecules with several residues (Val279, Gly342, Ile344, Arg378, and Arg379). These interaction patterns suggest that both **33** and **34** bind stably to the ACLY allosteric site with high affinity, which is consistent with their potent enzymatic inhibition. In a high-fat diet-induced MASH mouse model, administration of either compound **33** or **34** was reported to alleviate hepatic steatosis, systemic insulin resistance, and oxidative stress^131^.

**Figure 11.**
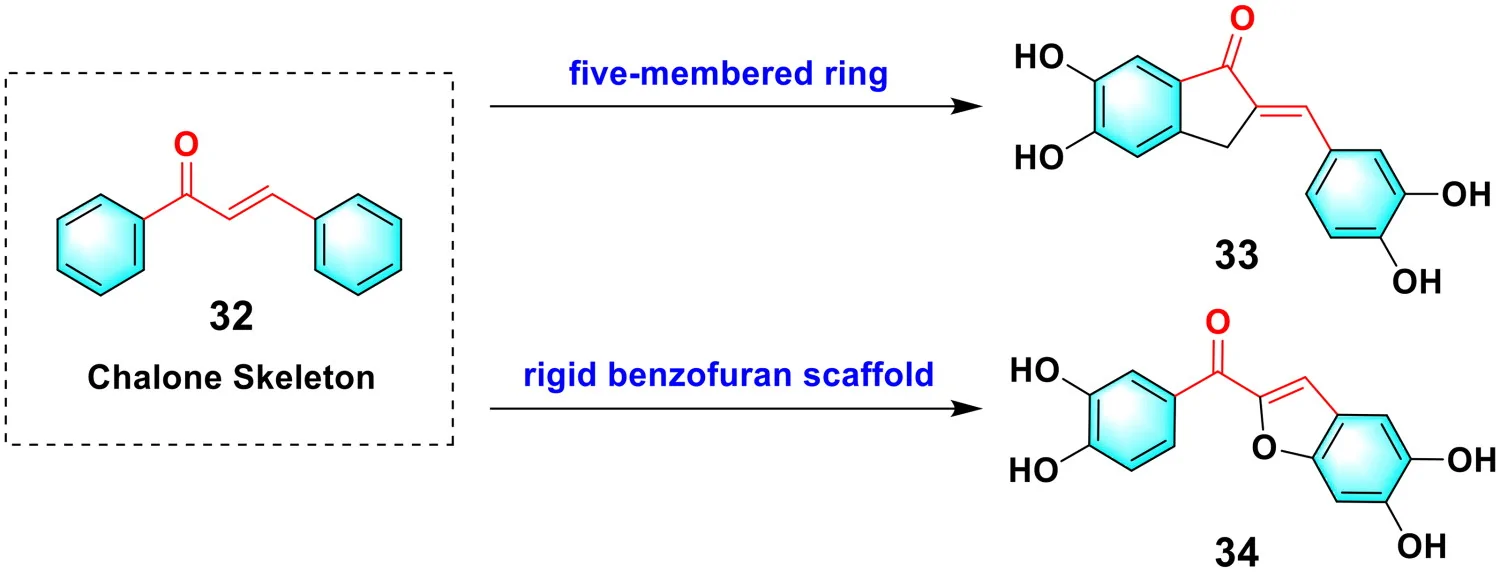
The chemical structures of chalcone skeleton analogues. Figure was created by the author using ChemDraw software, based on reference^131^.

### Other ACLY inhibitors

In 1993, Chan et al. isolated the alkaloid purpurone (compound **35**, Figure 12) from the marine sponge Iotrochotasp. It was shown to have an IC_50_ of 7 μM in a coupled enzyme assay, to inhibit ACLY activity in a dose-dependent manner, to reduced fatty acid synthesis in HepG2 cells, and to show no cytotoxicity^132^.

**Figure 12.**
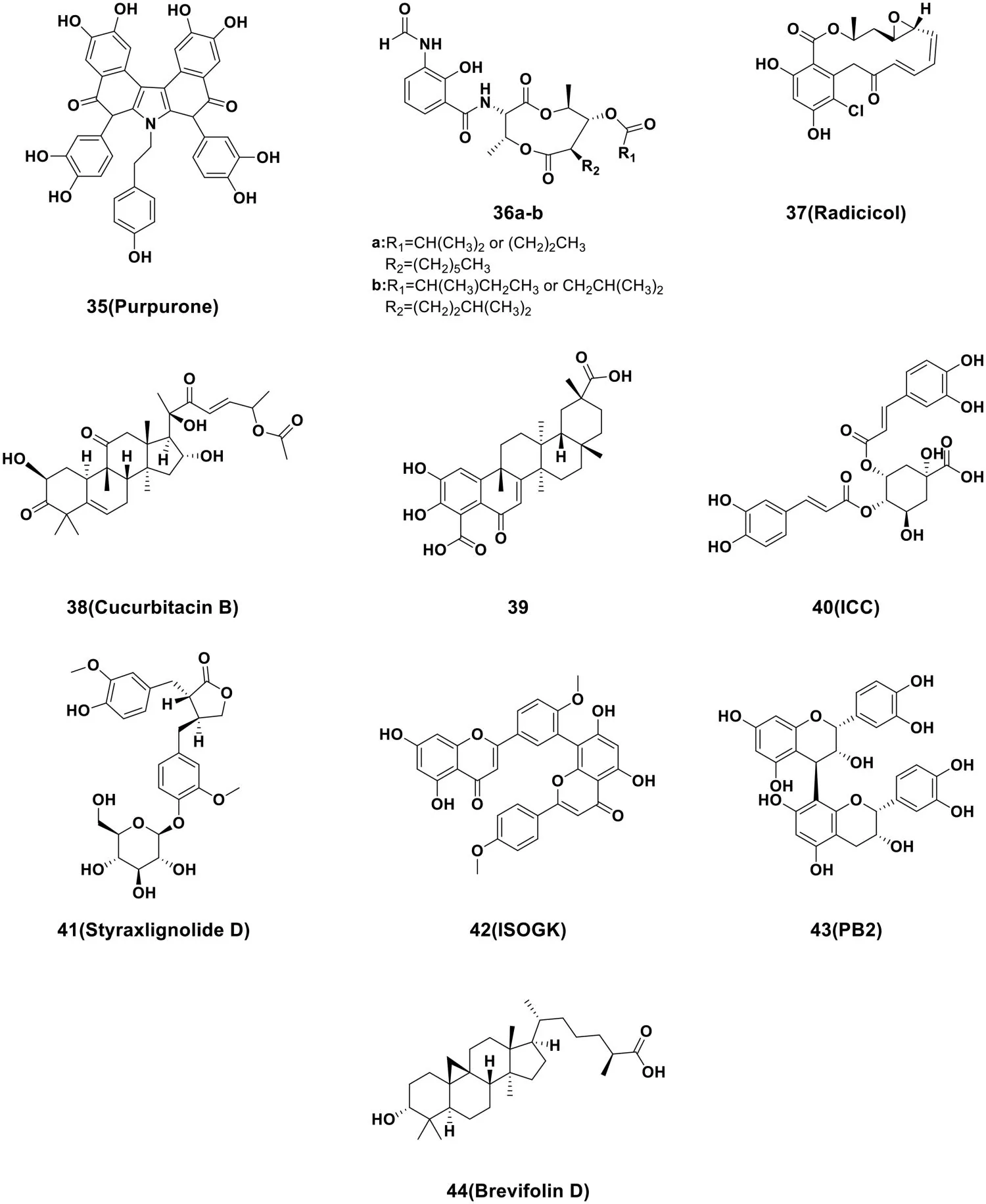
Other chemical structures of ACLY inhibitors from natural products. Figure was created by the author using ChemDraw software, based on references^129^^,^^132^^–^^140^.

In 1997, Barrow et al. discovered a series of antimycin-type ACLY inhibitors from Streptomyces. These feature a unique 9-membered dilactone ring linked to a 3-carboxamide salicylamide via an amide bond. The hydrophobic side chains at C-7 and C-8 were critical for inhibition, with compounds **36a** and **36b** showing K_i_ values of 4.2 and 4.0 μM, respectively^133^.

In 2000, Ki et al. isolated radicicol (compound **37**) from Monosporium bonordenand identified ACLY as a target via affinity chromatography. Enzymatic kinetics characterised it as a non-competitive inhibitor with K_i_ values of 13 μM (vs. citrate) and 7 μM (vs. ATP)^134^. Radicicol binds to an allosteric site, and its resorcylic acid lactone macrocycle is proposed to inhibit activity by altering conformation.

Cucurbitacin B (compound **38**), a tetracyclic triterpenoid from cucumber, inhibited the proliferation of prostate cancer cells PC-3 and LNCaP with an IC_50_ value of approximately 0.3 μM, while sparing normal prostate epithelial cells^135^. Mechanistic studies suggested that it acts by inhibiting ACLY phosphorylation; notably, ACLY overexpression attenuated its pro-apoptotic effect, supporting ACLY as a direct target. The α, β-unsaturated ketone in its scaffold may mediate covalent or hydrophobic interactions. Furthermore, triterpenoid compound **39** (IC_50_ = 0.44 μM) from Tripterygium wilfordii adds to the pool of natural ACLY inhibitors. SAR analysis suggested the essential roles of the C-3 hydroxyl and the conjugated A-ring system^136^.

Among phenolic acid inhibitors, dicaffeoylquinic acid derivatives show notable activity. Isochlorogenic acid C (compound **40**, ICC) from Acanthopanax senticosus had an IC_50_ of 0.14 μM. Its binding to ACLY was confirmed by cellular thermal shift assay (CETSA), and enzyme kinetics classified it as non-competitive. SAR studies suggested that the ortho-dihydroxyphenyl moiety is an important feature, potentially forming hydrogen bonds with polar residues in the allosteric pocket^129^. In 2024, Li et al. isolated lignans from Pouzolzia zeylanica; among them, styraxlignolide D (compound **41**, IC_50_ = 7.62 μM) formed a strong hydrogen bond with the catalytic residue Glu599 and reduced total cholesterol in HepG2 cells^137^.

Regarding flavonoid inhibitors, the Isoginkgetin (compound **42**, ISOGK, IC_50_ = 5.46 μM) was shown to bind directly to ACLY by surface plasmon resonance (SPR) and CETSA. In animal models, it was reported to lower blood lipids and attenuate atherosclerosis. Critically, its lipid-lowering effect was reduced upon hepatocyte-specific ACLY knockdown, supporting ACLY-dependent efficacy^138^. Proanthocyanidin B2 (compound **43**, PB2), a B-type dimer from grape seeds (IC_50_ = 2.73 μM), is a non-competitive inhibitor whose multiple phenolic hydroxyls are proposed to form hydrogen bonds and π–π stacking interactions at the allosteric site, suggesting a mechanism for the hypolipidemic effects of grape seed extracts^139^. Brevifolin D (compound **44**), a cycloartane triterpenoid, is an ACLY/ACC1 dual inhibitor isolated from the endangered plant short-leaved Pseudotsuga (ACLY IC_50_ = 9.6 μM; ACC1 IC_50_ = 11.0 μM). Its C-27 carboxylic acid group forms critical hydrogen bonding interactions with Gly-664, His-760, and Glu-599 of ACLY, and also participates in the binding of ACC1. This compound represents a new structural class of ACLY inhibitors from natural sources^140^.

## Discussion and conclusion

ACLY catalyses the cleavage of citrate to generate acetyl-CoA, coupling glucose metabolism with lipid synthesis and histone acetylation, and has been recognised as an important hub linking cellular metabolism and epigenetic regulation. This review systematically summarises the structure, catalytic mechanism, and functional regulation of ACLY under both normal and pathological conditions, and further outlines strategies for the design and development of ACLY-targeted drug molecules. In various pathological states, including metabolic disorders, immune-mediated inflammatory diseases, cancer, and neurodegenerative diseases, ACLY often exhibits increased upregulation in expression or activity, supporting the biosynthetic demands of rapidly proliferating cells, driving inflammatory responses, or contributing to neuropathological progression. The acetyl-CoA produced by ACLY possesses dual roles as both a metabolic substrate and an epigenetic regulator, making ACLY an attractive therapeutic target in these disease fields.

Although ACLY inhibition holds therapeutic promise across multiple diseases, its clinical translation has been hampered by compensatory pathway activation and tissue-restricted prodrug activation. Upon ACLY inhibition, compensatory enzymes such as ACSS2 can restore acetyl-CoA supply from alternative carbon sources, suggesting that isolated ACLY blockade may be insufficient to fully deplete the acetyl-CoA pool. Current efforts have therefore shifted towards multi-targeted strategies, such as EVT0185 (dual ACLY/ACSS2 inhibition) and 326E (synergistic ACLY/PPARα modulation), though further optimisation of their structures and pharmacological properties is needed. Additionally, currently marketed and clinically investigated ACLY inhibitors (e.g., ETC-1002 and 326E) rely on liver-specific ACSVL1-mediated activation, which may limit their efficacy outside the liver. Therefore, developing novel ACLY inhibitors that do not depend on tissue-restricted activation represents a key direction for future research.

## Data Availability

The authors confirm that the data supporting the findings of this study are available within the article.
